# Supplementary material for: A cytosolic surveillance mechanism activates the mitochondrial UPR
Source: Nature. 2023 Jun 7;618(7966):849–54. doi: 10.1038/s41586-023-06142-0 (PMC10284689; doi:10.1038/s41586-023-06142-0)
Supplement: Supplementary file 1 — This file contains Supplementary Figs. 1–3 and Table 4. [file 41586_2023_6142_MOESM1_ESM.pdf]

nature portfolio

https://doi.org/10.1038/s41586-023-06142-0

Supplementary information

A cytosolic surveillance mechanism activates the mitochondrial UPR

In the format provided by the authors and unedited

Supplementary Figure 1. Uncropped gel scans. Cropped version of the gel images are indicated with the dotted line.

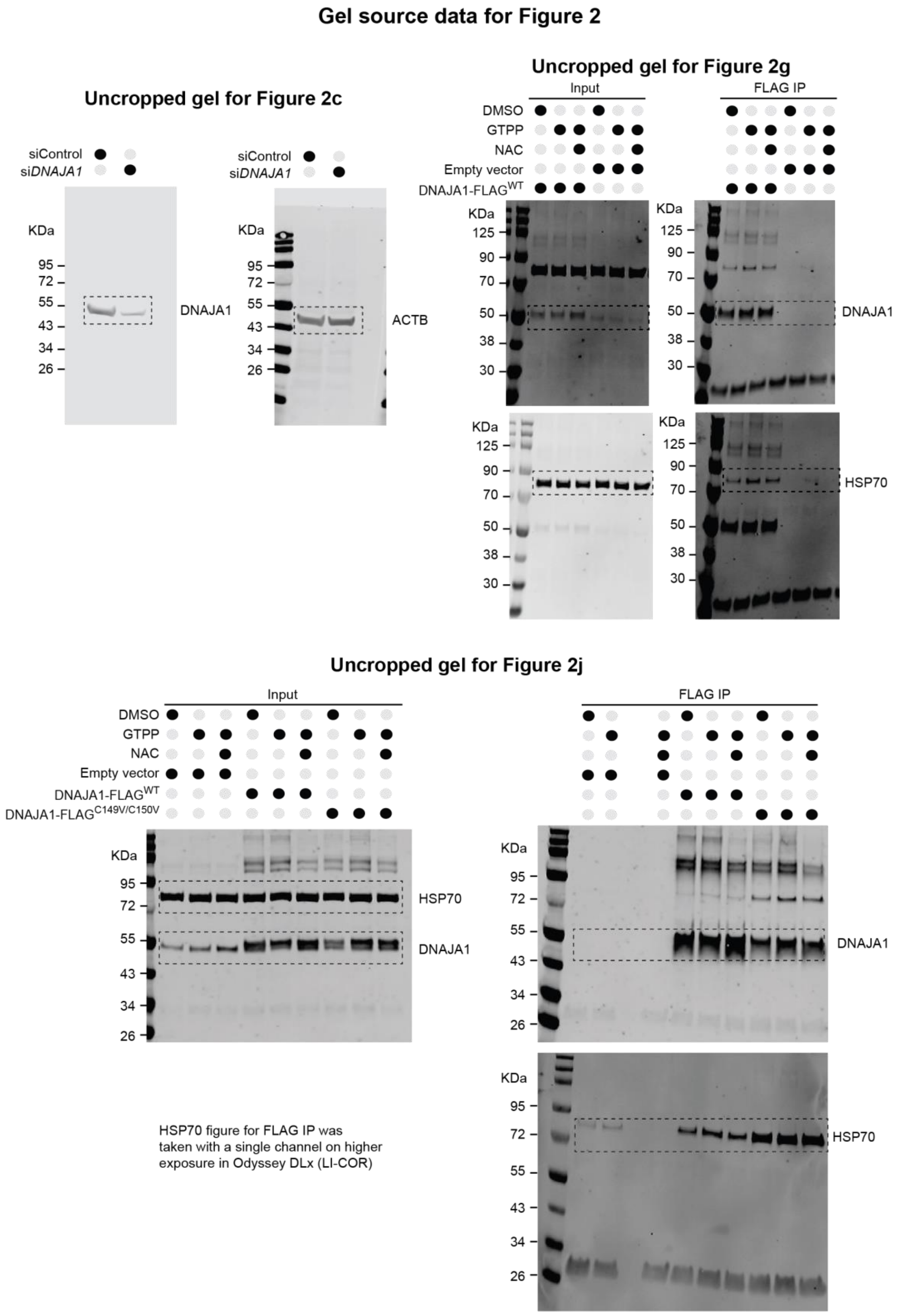

Gel source data for Figure 3

Uncropped gel for Figure 3a

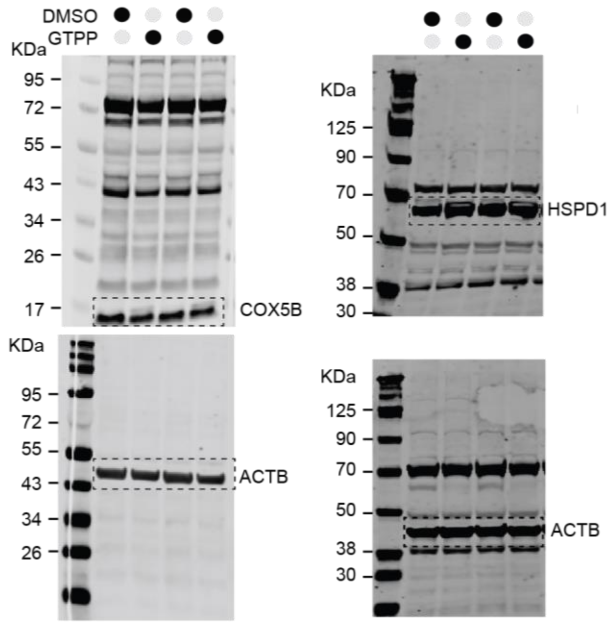

Loading controls were run in separate gels

Uncropped gel for Figure 3e

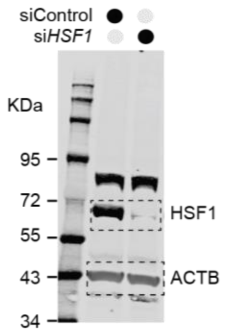

Uncropped gel for Figure 3h

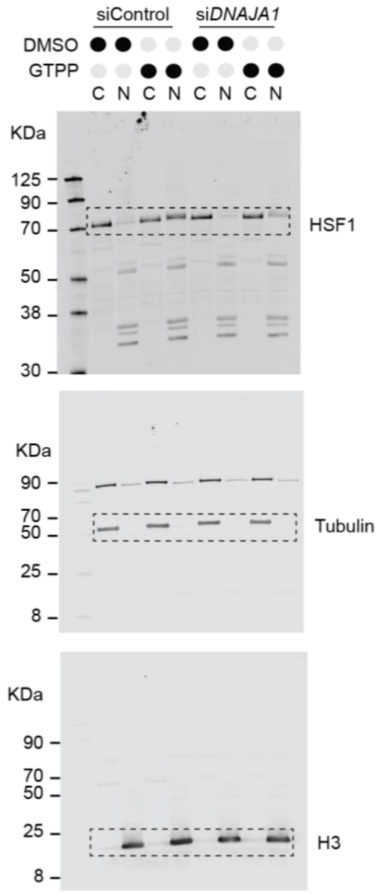

Uncropped gel for Figure 3f

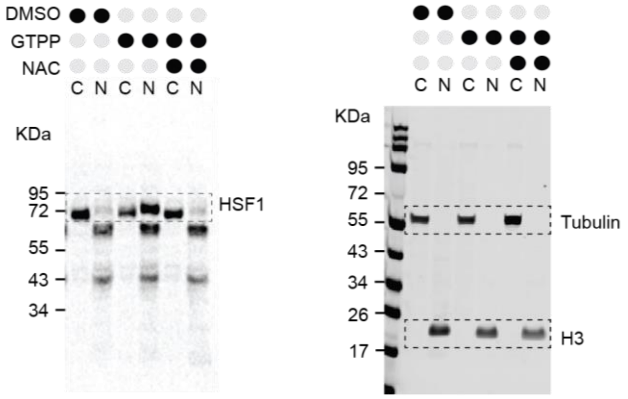

Gel source data for Figure 4

Uncropped gel for Figure 4m

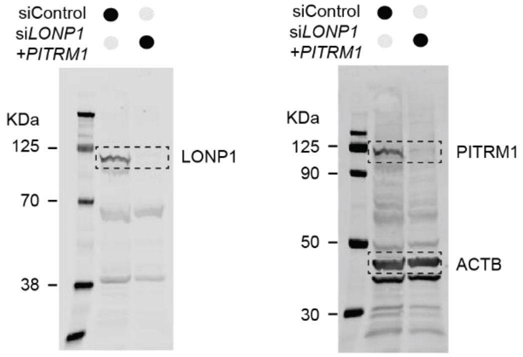

Gel source data for Extended Data Figure 1

Uncropped gel for Figure 1e

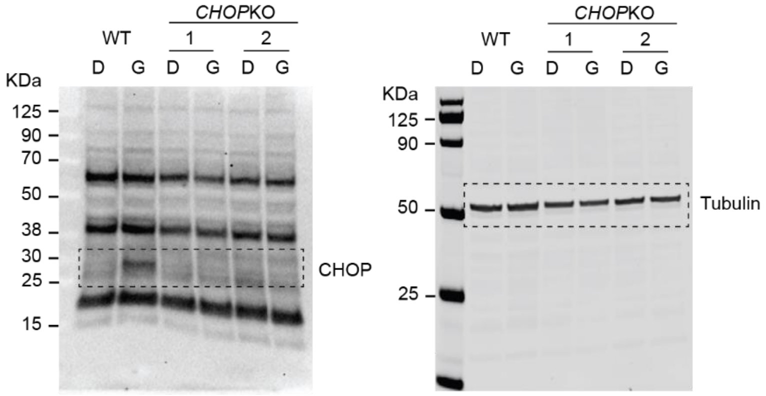

Uncropped gel for Figure 1f

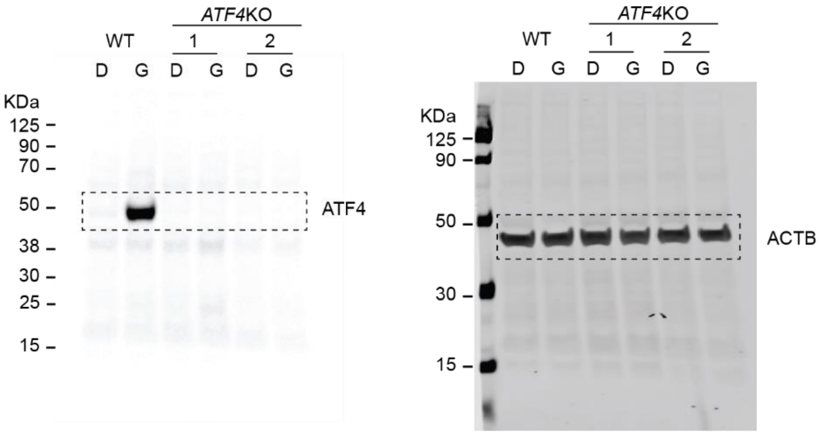

Gel source data for Extended Data Figure 6

Uncropped gel for Figure 6a

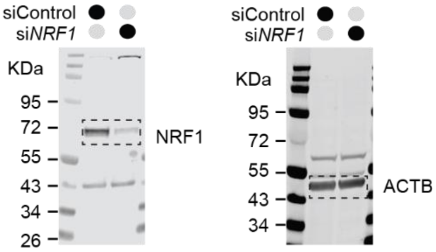

Gel source data for Extended Data Figure 7

Uncropped gel for Figure 7a

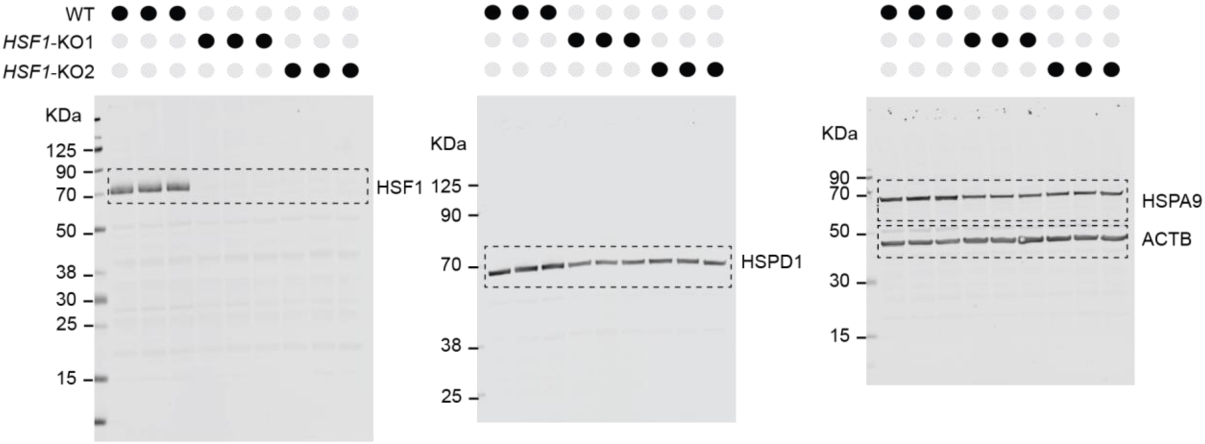

Uncropped gel for Figure 7f

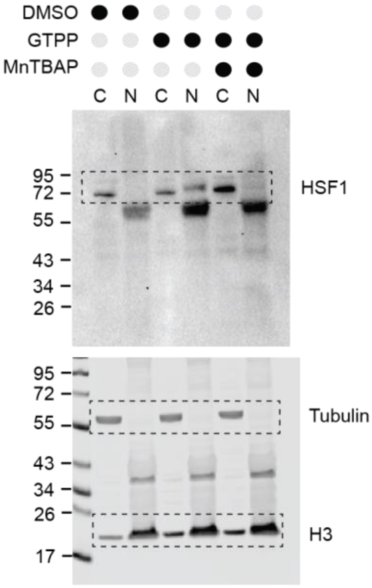

Uncropped gel for Figure 7g

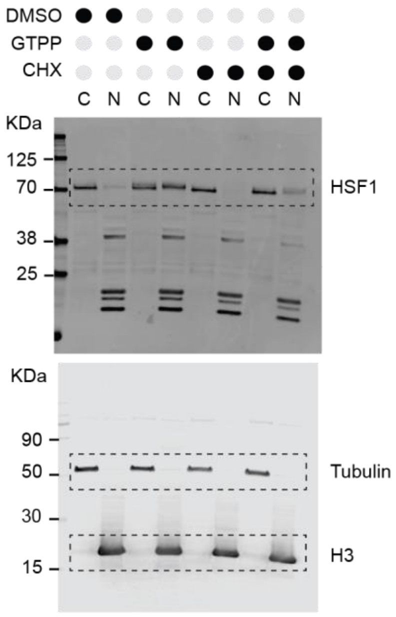

Uncropped gel for Figure 7i

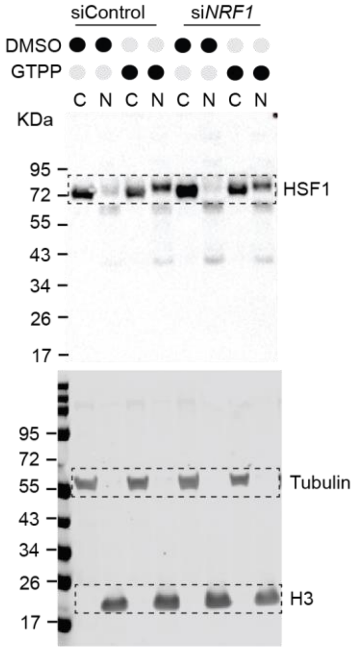

Uncropped gel for Figure 7k

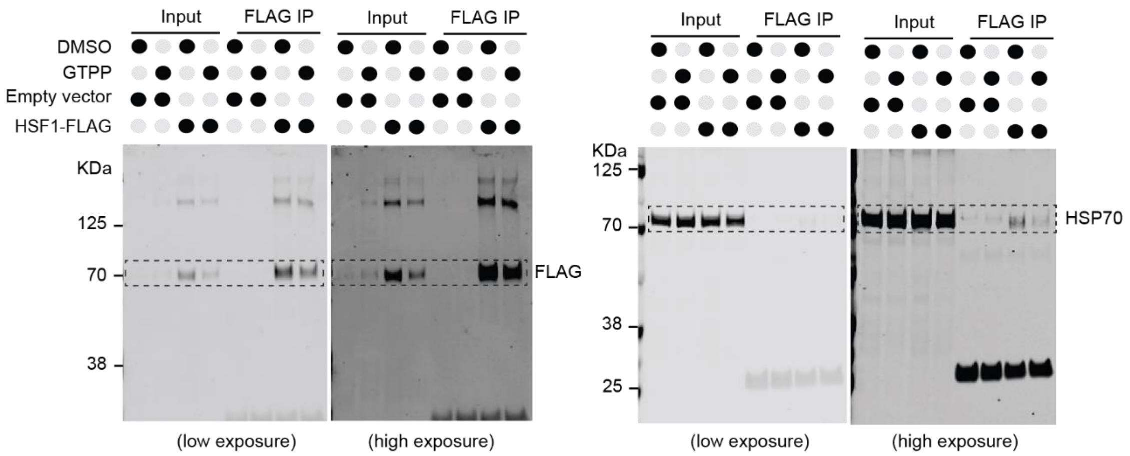

Gel source data for Extended Data Figure 9

Uncropped gel for Figure 9e

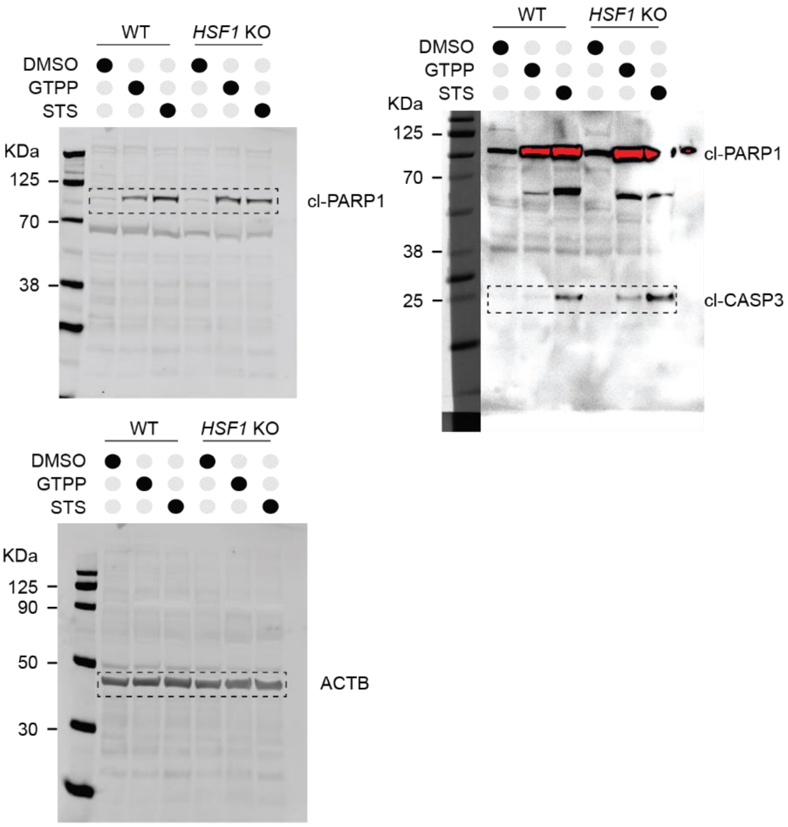

Gel source data for Extended Data Figure 10

Uncropped gel for Figure 10b

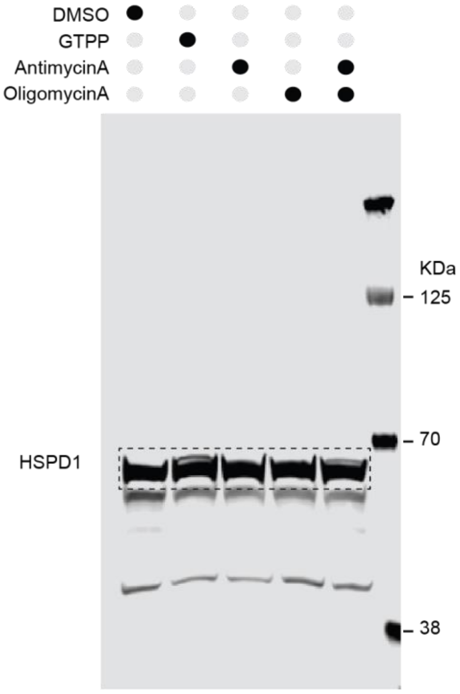

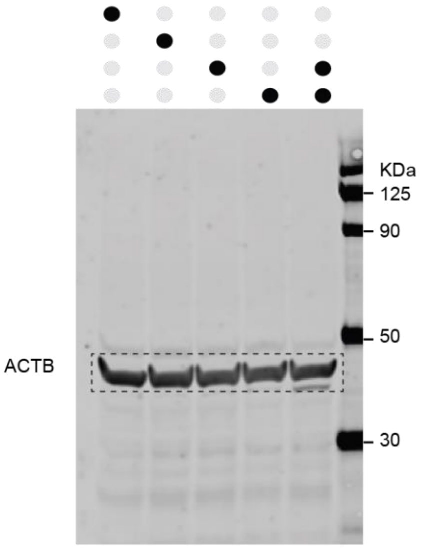

Uncropped gel for Figure 10k

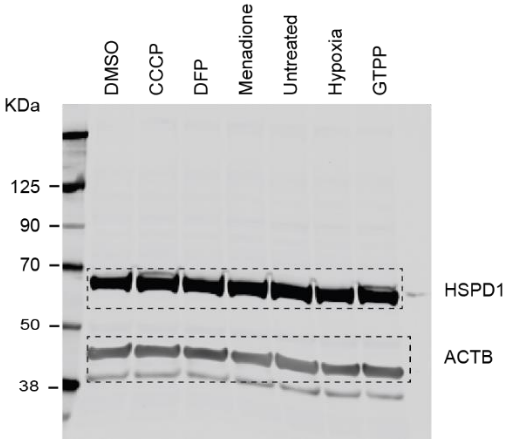

Supplementary Figure 2. All gel images used for quantification.

Supplementary Information for Figure 2h

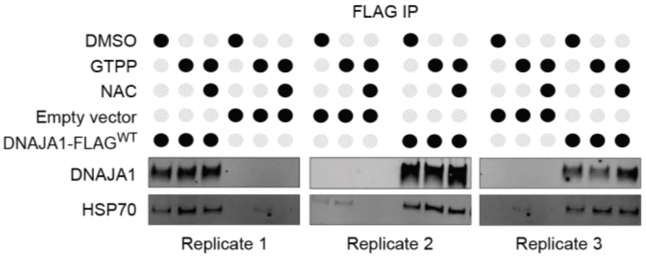

Supplementary Information for Figure 3g

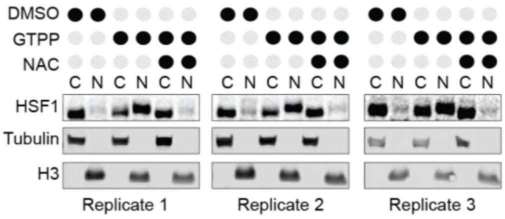

Supplementary Information for Figure 3i

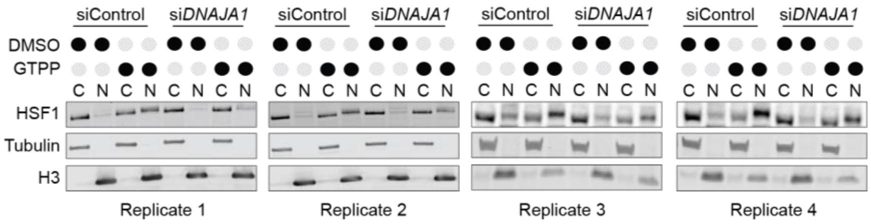

Supplementary Information for Extended Data Figure 7h

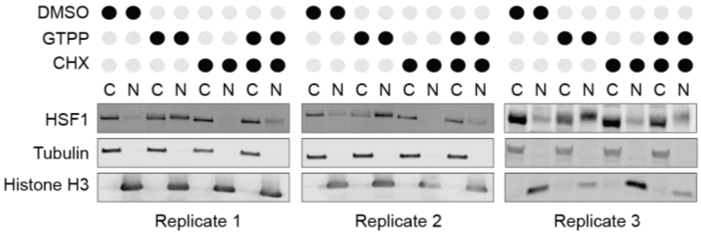

Supplementary Information for Extended Data Figure 7j

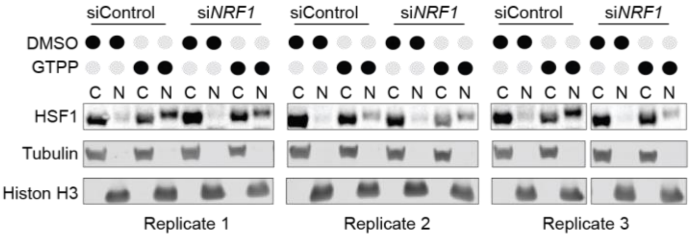

Supplementary Information for Extended Data Figure 7l

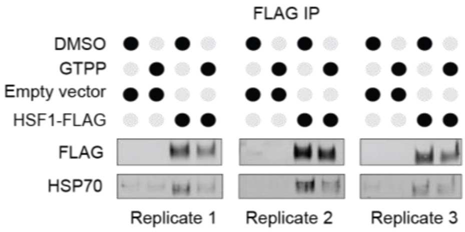

Supplementary Figure 3. Examples for gating strategy of flow cytometry analysis.

Gating example for ROS measurement with MitoSOX (Fig. 1c, Extended Data Fig. 2f, g, i, k, m, 10a, j)

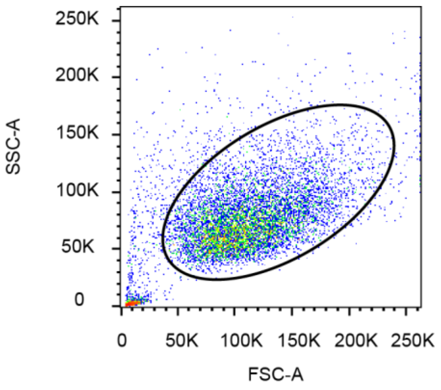

Gating example for cell death measurement with Alexa Fluor 488 Annexin V (Extended Data Fig. 5f)

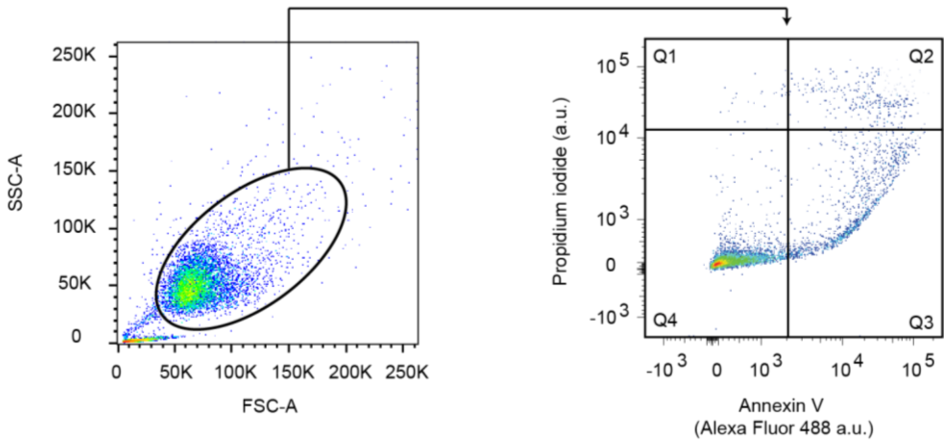

Supplementary Table 4. Primer list for qPCR analysis

Target gene	Direction	Sequence
HSPD1	fwd	CGTCTTGAATAGGCTAAAGG
	rev	TGAACGTCTTCAAGATTCAG
HSPE1	fwd	TTCTAAAGGAAAGGGTGGAG
	rev	TCAGTCTACGTACTTTCCAAG
LONP1	fwd	GAGACCAATATTCCTAAGCG
	rev	CTTGATGATCTTTAGCTGCTC
HSPA9	fwd	CAATGGGGATACCTTCTTAG
	rev	ATAGGGCAAATTGATGTCAG
ATF4	fwd	CCTAGGTCTCTTAGATGATTACC
	rev	CAAGTCGAACTCCTTCAAATC
NDUFA4	fwd	AATTTTTAGCTTAGGGCCTG
	rev	ATACAAAGAGGGGGATCAAG
UQCRC2	fwd	GTGAGTCATCCTGTTCTAAAG
	rev	CATTTCTGTTCTCGGATTTCAC
SDHA	fwd	AGCATGCAGAAGTCAATG
	rev	ATTTTCCCACAACCTTCTTG
DDIT3	fwd	CTTTTCCAGACTGATCCAAC
	rev	GATTCTTCCTCTTCATTTCCAG
DNAJA2	fwd	ACAGGAACAAAACATAACCC
	rev	TTCGAGGCACTCCTAATATC
DNAJB1	fwd	TTAAGAGAGATGGCTCTGATG
	rev	CTTTGAATACGACGGGTATC
NRF1	fwd	CCACACATAGTATAGCTCATC
	rev	TACCAACCTGGATAAGTGAG
HSPA1A	fwd	AATTTCCTGTGTTTGCAATG
	rev	AAAATGGCCTGAGTTAAGTG
ATF5	fwd	CATGGAGTCTTCCACTTTC
	rev	GGAGTGACATGGCTGTAG
TRIB3	fwd	GACCGTGAGAGGAAGAAG
	rev	GAGTATCTCAGGTCCCAC
CHAC1	fwd	TGAAGATCATGAGGGCTG
	rev	CGCAGCAAGTATTCAAGG
